# Comparative Review of Halal Certification Frameworks for Poultry Meat in Malaysia, Singapore, and Indonesia

**DOI:** 10.3390/foods15040659

**Published:** 2026-02-11

**Authors:** Bo-Zheng Zhang, Ji-Woon Moon, Jung-Min Park

**Affiliations:** Department of Food Marketing and Safety, Konkuk University, Seoul 05029, Republic of Korea; slurpee24@163.com (B.-Z.Z.); nym21@naver.com (J.-W.M.)

**Keywords:** Islamic food standards, food industry regulations, comparative analysis, ASEAN, standardization

## Abstract

The term “halal” is derived from the Arabic word for “permitted” or “allowed”. Issues arise in the global halal industry regarding the implementation of halal standards due to differences between Islamic countries. Producers find it challenging to comply with the different halal standards, thereby complicating international trade in halal products. Using a qualitative approach, this study employed a literature review and document analysis approach, utilizing Scopus for literature and meticulously examining official halal standard documents from bodies such as JAKIM, MUI, and MUIS. In Malaysia, the Malaysian Veterinary and Religious Authority (JAKIM) oversees halal awareness programs and regulates food production facilities in accordance with the standards documented in the Malaysian Halal Management System 2020, Malaysia Halal Certification Procedure Manual (2014 and 2020), and MS 1500:2019 (Halal Food). In Indonesia, the Halal Guarantee System (HAS) is implemented in accordance with the Halal Fatwa Issuing Institution (MUI) and Halal Product Assurance Organizing Agency (BPJPH) regulations through HAS 23:10:3:2012. Singapore uses MUIS-HC-S001 and MUIS-HC-S002. This study examines and compares halal standards practiced in these three Islamic countries to improve the performance of the global halal food industry and facilitate international trade.

## 1. Introduction

The global halal food industry is growing rapidly, providing halal food producers with new opportunities to expand their domestic and international markets. The rise in health concerns has led to a greater acceptance of halal food, as it encompasses the concept of consuming clean and hygienic food for better health [1]. Consumers are becoming more health conscious, which influences not only their physical health but also their mental state and quality of life. Furthermore, non-Muslim consumers are also responding positively to halal food certification [2].

Islam is currently the fastest-growing religion worldwide, with an annual growth rate of 1.5%. There are currently approximately 2.061 billion Muslims worldwide, and it is projected that this population will account for 26.4% of the global population by 2030 [3]. The halal food trade has grown due to an increase in the global Muslim population, which is expected to reach three billion people. The halal food market is currently valued at USD 2.86 trillion and is projected to reach USD 4.177 trillion by 2028 [4,5,6].

In the late 1980s, the certification and production of halal foods began to increase [7] to penetrate the Southeast Asian and Middle Eastern markets. Ironically, non-Muslim countries such as New Zealand, the United Kingdom, and the United States export most halal food products, accounting for approximately 90% of the global halal market. This is particularly evident in the meat industry [8].

The halal meat industry is driven by fast-food restaurants and specialty butchers, and processed meat products are purchased by consumers worldwide [9]. The global processed meat market was valued at USD 1.567 trillion in 2022 and is expected to grow further by 2030 [10]. Within this broad market, the demand for halal-certified chicken products is particularly high in Malaysian communities [11,12], reflecting a significant segment of the global halal processed meat market. To be considered halal, meat and poultry must be slaughtered in accordance with Islamic rituals. Dhabihah, the traditional Islamic slaughtering method, involves a Muslim slaughterer reciting the name of God (Tasmiyah) while using a sharp instrument to slit the animal’s trachea, esophagus, and carotid artery. The purpose of this ritual is to ensure hygienic purity through complete bloodletting, minimize animal suffering, and realize religious sanctity and ethical mercy. This means that all materials used in food production should be halal, and facilities, utensils, tools, and packaging must not be contaminated with non-halal elements such as pork, alcohol, or impure substances. Halal is an Arabic phrase that means allowed or permitted by Islamic law. Another popular term is halalan toyyiban, which merely means permissible for consumption with respect to Sharia law, if the food is safe and not harmful. The terms non-halal and haram, in contrast, refer to foods and items that are forbidden or prohibited [13,14,15,16]. In Muslim countries, food safety and halal principles are inherently harmonious, as halal food is fundamentally required to be safe, wholesome, and hygienic. As a simple description, halal foods are permissible for consumption with relation to Sharia law if they are safe and not harmful. In addition to fulfilling Sharia law, which is a requirement for Muslims, the food safety factor plays a significant role in determining the toyyiban; one example is the wholesome (safety, cleanliness, nutrition, and quality) aspects of the food. However, most consumers who prefer halal meat products are unaware of the principles of the halal supply chain. Instead, consumers of halal meat are typically buying foods bearing a halal mark on the packaging to help them make informed choices [17,18].

Halal is currently an essential issue in food production, as it is required to access the markets of Islamic countries. With the rapid growth of the global Muslim population, the mark indicating that food is halal has become an important indicator of quality, safety, and consumer trust in the international food market. Halal management is governed by halal regulations in various countries, particularly those related to certification and labeling [19]. Food producers must understand and implement halal standards related to materials, products, facilities, and management systems to obtain halal certification [20]. Consequently, halal certification plays a critical role in facilitating global food trade and ensuring regulatory compliance. From a management system perspective, halal policies and standard operating procedures must be implemented.

Unfortunately, producers often fail to meet halal standards because of a lack of understanding of halal requirements and import regulations, as well as ignorance and confusion. This can include the misuse of halal certification, contamination during production, use of animals that have not been slaughtered according to rituals, and disregarding animal welfare [21,22]. Additionally, the complexity of the food production chain, numerous halal-related issues, and fraud concerning food labeling have become significant problems.

In addition, halal standards vary globally, which have substantial implications. Multiple Halal Certification Bodies (HCBs) can approve food products but often prioritize national regulations over international alignment. From an estimated 300 halal certification bodies worldwide, approximately 120 are officially registered as active HCBs, meaning that they hold formal accreditation or Mutual Recognition Agreements with governmental bodies [23,24].

In light of the growing demand for halal food, which is essential for Muslims and is slowly gaining popularity among non-Muslims, this study specifically aims to discuss and analyze halal certification and food legislation pertaining to poultry products. The countries studied were Majlis Ugama Islam Singapore, Majelis Ulama Indonesia, and Jabatan Kemajuan Islam Malaysia. This selection was made because halal standards in the poultry food industry differ significantly among Islamic countries, and there are inherent limitations to achieving full halal standardization due to varying halal criteria regulations across nations and the application of non-halal standards in certain contexts.

To address these complexities and contribute to a more unified understanding of halal certification within the poultry sector, this study addressed the following research questions:

What are the key components and procedural characteristics of halal poultry certification and regulatory frameworks in Malaysia, Indonesia, and Singapore?

What are the main regulatory bottlenecks and significant procedural discrepancies in halal standards (including audit methods, slaughter processes, labelling, and import/quarantine procedures) observed in the cross-border halal poultry trade among Malaysia, Indonesia, and Singapore?

What implications can be drawn from the comparative analysis of halal standards and regulations across these three countries to enhance the performance of the global halal poultry industry and facilitate international trade?

Ultimately, this study provides foundational data for halal standardization and guidelines to help improve the performance of the global halal poultry industry; these guidelines are also expected to improve access to countries that export halal poultry products.

## 2. Materials and Methods

### 2.1. Study Design

This study employed a descriptive, comparative document analysis design, following a narrative review approach. The primary objective was to systematically identify, analyze, and synthesize the regulatory frameworks governing halal certification for poultry meat in Malaysia, Singapore, and Indonesia.

### 2.2. Data Collection

The search strategy involved multiple avenues:

Electronic Databases: Scopus, Google Scholar, and Web of Science were searched using keyword combinations such as (“halal certification” or “halal standard*”) and (“poultry” or “chicken meat” or “broiler”) and (“Malaysia” or “Singapore” or “Indonesia”). The search focuses on obtaining the latest versions of regulations and standards while also covering the key legal documents that form the foundation of the regulatory systems in various countries.

Official Institutional Websites: The primary websites of key regulatory and certification bodies were systematically reviewed to obtain primary legal documents, standards, manuals, and guidelines. These included:

1. Malaysia: Jabatan Kemajuan Islam Malaysia (JAKIM), Department of Standards Malaysia (JSM), Department of Veterinary Services (DVS);
2. Singapore: Islamic Religious Council of Singapore (MUIS), Singapore Food Agency (SFA);
3. Indonesia: Badan Penyelenggara Jaminan Produk Halal (BPJPH), Majelis Ulama Indonesia (MUI), Lembaga Pengkajian Pangan, Obat-obatan, dan Kosmetika (LPPOM MUI).

### 2.3. Document Screening and Inclusion Criteria

Documents were included based on the following criteria:

1. Type: Official government regulations, acts, standards, certification procedure manuals, and technical guidelines directly related to halal certification or food safety for poultry/meat products;
2. Relevance: Documents explicitly addressing certification processes, slaughter requirements, import/export procedures, labeling, sanitary standards, or organizational structures for halal assurance in the three target countries;
3. Source: Published by national government agencies, recognized religious authorities, or international bodies (e.g., Codex). Peer-reviewed academic literature that provided analysis of these frameworks was also included;
4. Language: Primarily English and Bahasa Indonesia/Malaysia documents.

Documents such as general news articles, non-official blog posts, and documents not specifically related to poultry meat or the core certification process were excluded.

### 2.4. Data Analysis and Synthesis Framework

Qualitative content analysis and thematic synthesis were employed Data extracted from the included documents were organized into pre-defined thematic categories common across the three countries: (1) Halal certification, (2) The process for registering and quarantine, (3) Malaysian import procedures, (4) Malaysia food labeling format, (5) Malaysian sanitary and quarantine standards, and (6) Organizations and regulations of halal certification.

Data within each theme were then compared across Malaysia, Singapore, and Indonesia to identify similarities, divergences, and potential bottlenecks. Key findings were synthesized into descriptive summaries and comparative tables (Table 1).

## 3. Results

Halal products circulating in Indonesia, Singapore, and Malaysia follow the halal standards of each country. This section examines the halal standards and certification systems in these countries. The findings can be used as a reference for recognizing halal standards for poultry and meat in other countries.

### 3.1. Malaysia

#### 3.1.1. Halal Certification in Malaysia

In Malaysia, halal certification is not mandatory but is widely used as a marketing tool, given that approximately 60% of the population is Muslim [28,29]. Due to the high standards of halal certification, non-Muslims also buy halal products because they are perceived as safe. Mandatory certification only applies to a few items specified by the Malaysian government. Halal certification arrangements in Malaysia, set out in Article 28 of the 2011 Trade Deed, remain voluntary. Although regulations that require all imported meat to be halal-certified have been enacted, state intervention is still limited to information (corridor information) and only applied to imported meat. Under the Slaughter Act 1975 [30], all meat products imported into Malaysia must be halal certified.

The halal certification procedure for slaughterhouses, as stipulated by JAKIM, involves a multi-stage process. It commences with the submission of a comprehensive application dossier by the overseas entity to the DVS. Following this, the DVS and JAKIM conduct a thorough review and inspection of the submitted documents to assess compliance with preliminary requirements. Subsequently, a mandatory on-site audit is performed at the applicant’s slaughterhouse and processing facilities to verify adherence to Malaysian halal and sanitary standards in practice. Only upon successful completion of all preceding stages does the authority proceed to grant formal approval and issue the halal certificate, thereby authorizing the export of poultry meat to Malaysia [31,32,33,34].

Slaughtering is also a part of the halal certification process, which is more demanding than for other food products. Details of the slaughtering process are presented in JAKIM’s Malaysian Protocol for Halal Food Production, Preparation, Handling, and Storage—General Guidelines (second revision) [25].

There are specific requirements that both slaughterers must be Muslim adults of sound mind and integrity, and they must hold a halal slaughter certificate issued by a recognized body. Animals must be slaughtered in a slaughterhouse designated by the Malaysian Veterinary and Religious Authority (JAKIM), and all meat must be labeled with the designated number of the slaughterhouse and meat-packing plant, date of production, and form of slaughter (i.e., to specify whether it is in accordance with Muslim law). To acquire halal certification for a slaughterhouse, overseas applicants must apply to the Department of Veterinary Services Malaysia (DVS) for the approval of their slaughterhouses and meat processing plants before applying for Malaysian halal certification (Table 2) [35,36].

#### 3.1.2. The Process for Registering and Quarantine

Meat products need to comply with meat hygiene and phytosanitary requirements prior to export and are subject to customs quarantine. The import of meat and poultry into Malaysia must be inspected and approved by the DVS and Islamic Development Authority (JAKIM). The process of registering a DVS facility includes submitting and reviewing documents, conducting offsite audits, and inspecting the facility. Once approved by the DVS, overseas slaughterhouses and meat processing plants are registered on the DVS’s website [37], and overseas applicants can apply for Malaysian halal certification.

The Malaysian Quarantine and Inspection Services (MAQIS) are responsible for quarantining imported food products. This process includes inspecting the contents of plants, animals, carcasses, fish, and agricultural products. Soil, microorganisms, and transport equipment are also inspected for pests, diseases, and contaminants to ensure that the import conditions are consistent with the issued permits, licenses, and certifications. Quarantine services are administered by Veterinary Inspection and Quarantine Services. The procedure involves applying for quarantine, paying a quarantine fee, and, if approved, receiving a veterinary quarantine certificate. Customs is then authorized and notified of the certification. Imported meat, meat products, dairy products, pork, and pork products are inspected by designated MAQIS livestock/food inspectors upon entry into the country. Applications can be made electronically for the electronic quarantine of animal products by using Dagang Net, an electronic customs service.

#### 3.1.3. Malaysian Import Procedures

All imports to Malaysia must enter the country through designated ports and airports. Customs clearance stages include shipping and transport, import customs clearance, and imported food quarantine. Hygiene certificates, import permits, and a halal export certificate are also required.

All imported goods must be declared on a Customs No. 1 form. The Malaysian government operates a national single window system to simplify customs clearance. Permit applications are submitted via the ePermit system and verified by a Malaysian Import and Export Food Quarantine Department officer. If approved, a permit is sent to the Customs Information System.

#### 3.1.4. Malaysia Food Labeling Format

Food products imported into Malaysia can be labeled in either Bahasa Malaysia or English. Translations into other languages may also be included. Labeling regulations, primarily governed by Food Regulations 1985 under Food Act 1983, prescribe the specific contents of the label, the form and method of labeling, expiry dates, and other information that cannot be displayed. The label indicates the product name, ingredients, net weight or volume, expiry date, storage instructions, and other information such as nutritional information, GMOs, and additives [38].

#### 3.1.5. Malaysian Sanitary and Quarantine Standards

Any stage in the food supply chain, from breeding farms to breeders to livestock farmers, distributors, slaughterhouses, meat processors, retailers, and consumers, is susceptible to contamination or the presence of forbidden elements. All meat products must be free from harmful substances, such as bacteria, pathogenic bacteria, viruses, toxins, and heavy metals, as well as pesticides and veterinary drug residues resulting from the excessive use of chemicals. Feed safety is also crucial because feed containing excessive levels of antibiotics and heavy metals significantly affects the growth rate, feed intake, and immunity of chickens. This should also be reflected in the strict compliance requirements of the halal system. Because these aspects have not yet been covered in detail by halal regulations, we must examine the regulations of each country to determine the scope of future halal certification.

Meat product standards in Malaysia include microbial and chemical analyses (heavy metals, pesticide residues, and veterinary drug residues). In microbial analysis, the total count must be lower than 10^6^ CFU/g, and the coliform count must be lower than 5 × 10^1^ CFU/g (Table 3). In the chemical analysis, in the case of heavy metals, the lead limit is 0.5 mg/kg; cadmium limit, 1 mg/kg; arsenic limit, 1 mg/kg; tin limit, 40 mg/kg; antimony limit, 0.05 mg/kg; and copper limit, 1 mg/kg (Table 4).

Pesticide residues have a maximum amount of three components, including carbaryl, malathion, and phoxim, and veterinary drug residues have 34 components (Table 5).

#### 3.1.6. Organizations and Regulations of Halal Certification

The Malaysia Halal Certification scheme is a collaboration between various organizations that work together to maintain halal integrity, promote industry growth, and monitor halal practice implementation. The government agencies involved in Malaysia’s imports and exports are:

- Jabatan Standard Malaysia (JSM): JSM is part of the Ministry of Science, Technology and Innovation. It contributes to the promotion of the halal industry by developing Malaysian standards for halal food (MS 1500).
- Excise Department and Royal Customs: This department is responsible for import and export duties, excise duties, sales taxes, service taxes, and motor vehicle taxes. They are governed by the Customs Act of 1967 and control matters related to the import and export of goods under 30 laws and regulations in conjunction with other national departments.
- Malaysian Quarantine and Inspection Services (MAQIS): MAQIS inspects animals, plants, and fish imported to Malaysia for pests and contamination. It also conducts quarantine operations to ensure compliance with Malaysia’s food safety regulations [34].
- Department of Veterinary Services (DVS): The DVS is responsible for livestock and birds imported into Malaysia and provides quarantine facilities. The import licensing of livestock is governed by Livestock Ordinance 1953, Livestock Act 1962, Livestock Import Regulations 1962, and Federal Livestock Quarantine Act 1984. Export certificates are required for livestock and birds entering Malaysia.

In the case of the regulation of halal certification, Malaysia’s standards for halal food are documented in MS 1500:2019 and focus on the production, preparation, storage, and supply of halal food. These regulations broadly classify halal requirements into four categories: matters relating to employees, raw materials, production processes, and storage and transportation. The halal standard has been in use since 2004 and was revised for the third time in early 2019. MS 1500 is applied, as well as other standard guidelines such as MS 1480:2007 (Food Safety Management Systems—Hazard Analysis and Critical Control Points [HACCP]) and MS 1514:2009 (General Principles of Food Hygiene) to strengthen the food production process, and MS 2200-2, MS 2400-1, MS 2565, and MS 2627 among others [39,40,41,42,43,44,45,46].

In addition to halal regulations, the Trade Act 2011, Food Act 1983, and Food Regulations 1985 govern food safety and hygiene management. The Animal Act 1953 (reviewed in 2006), Regulation of Animals Act 1962, Slaughterhouses Act 1993, and Customs Act 1967 apply to the management of imported meat [47,48,49,50,51,52,53].

### 3.2. Singapore

#### 3.2.1. Halal Certification in Singapore

The Majlis Ugama Islamah Singapura (MUIS), the sole halal certification body under Singapore’s Administration of Muslim Law Act (AMLA), is a national organization and one of the world’s top three recognized halal certification bodies. This organization is responsible for certifying halal food and products in Singapore under AMLA (Sections 88(1) and 88(2)) [54,55]. It is regulated by the AVA, which is responsible for agricultural and livestock production; however, with the establishment of the Singapore Food Agency (SFA) in April 2019, the AVA regulatory functions were transferred to the SFA [56]. MUIS is known for its fast and transparent process, along with Indonesia’s MUI and Malaysia’s JAKIM.

Since 1978, MUIS halal certification has focused on the Halal Quality Management System (HalMQ) [57] to issue halal certificates. Seven types of halal certifications are available for eating establishments, endorsements, food preparation areas, poultry abattoirs, products, storage facilities, and whole plant schemes (Table 6). The MUIS manages the certification system and is responsible for testing and issuing halal certificates to institutions. Additionally, Warees Halal is an organization that helps to maintain halal management systems. MUIS’s halal certification mark is globally recognized. Since 1978, MUIS has issued halal certifications in Singapore, adopting a voluntary approach to implementation [58,59].

In Singapore, the MUIS halal certification process follows a structured sequence. It initiates with an initial enquiry and preparation phase, where prospective applicants familiarize themselves with requirements. The formal application is then submitted electronically via the national GoBusiness Licensing Portal. Upon receipt, MUIS enters a processing phase, which encompasses administrative checks, document evaluation, and scheduling of necessary assessments. The certification stage involves the final review and issuance of the halal certificate upon satisfactory compliance. Following certification, a post-certification phase includes ongoing supervision, compliance monitoring, and handling of any amendments. Finally, the certificate enters a renewal cycle, requiring re-assessment before expiry to ensure continued adherence to halal standards [60,61].

Meat products are considered “high-risk”, and halal certification is obtained for products such as beef, mutton, and poultry that are supplied fresh, refrigerated, or frozen. The following meat and meat-based products are included: poultry and poultry-based items, beef extracts, beef tallow, chicken skin, chicken oil, flavorings, and gelatin. MUIS halal-certified meat processors and distributors supply fresh, chilled, and processed meat products to supermarkets and other retailers.

#### 3.2.2. The Process for Registering and Quarantine

To import meat into Singapore, one must apply for trade account activation (https://www.tradenet.gov.sg/tradenet/login.jsp, accessed on 5 February 2026) and import/export permits from the SFA. The required documents are the Singapore corporation’s UEN (Unique Entity Number) and CorpPass (corporate digital identity), as well as the HS code. A Customs Permit (often referred to in the process) can be obtained after applying for trade account activation through Singapore Customs and receiving approval. A related license must be obtained according to the SFA regulations. In general, all food products are subject to inspection and can be submitted for inspection via the SFA Inspection & Laboratory e-Service. Currently, submission requirements include a cargo clearance certificate, health certificate, related documents, and the products to be inspected.

To obtain a poultry product import license, an application must be submitted through the GoBusiness licensing system. All customs duties must be paid electronically via the TradeNet system using the Giro application. The application documents include institutional and product information, export history, quarantine certificates, factory floor plans, water supply information, employee information, information about the meat processing facility, videos of the facilities, and information about the Singapore import company.

#### 3.2.3. Singapore Imports Procedures

All food products imported into Singapore are subject to the Sales of Food Act and Food Regulations. All processed foods must be registered with the SFA, and importers must register their imports on the TradeNet system to obtain import permits. Singapore’s food safety requirements align with international standards, such as HACCP and the Codex Alimentarius Commission’s standards for food sanitation. Singapore distinguishes between import permits and registrations to ensure food safety. In the case of poultry, imports are permitted only by countries and facilities approved by the SFA.

The process of clearing imports into Singapore involves the following steps: import declaration, payment of duties, inspection and quarantine, and release of goods. Singapore’s current food regulations have been modified to suit domestic conditions and requirements based on internationally accepted import regulations and standards. These import regulations encompass a comprehensive framework, including mandatory health certificates for each consignment, accreditation of overseas farms and establishments for higher-risk products, and adherence to specific processing standards for items like dairy and infant formula. The SFA employs a science- and risk-based approach to ensure food safety and minimize foodborne illness risks for all imported products. Import licensing procedures maintained by the SFA are enforced for food safety, public health, and animal health reasons, not to restrict the quantity or value of imports. These procedures are statutorily required and published in government gazettes. Generally, all persons, registered firms, and institutions are eligible to register with SFA and apply for licenses/registrations.

Before importing food products, the following items must be confirmed.

1. Obtain a trader’s license or register with the SFA.
2. Comply with relevant food legislation.
3. Meet SFA conditions for specific types of food.
4. Satisfy SFA’s labeling requirements.
5. Apply for an import permit.
6. The relevant food registration needed to be completed.

#### 3.2.4. Singapore Food Labeling Format

Food labeling in Singapore is strictly regulated under the Sale of Food Act 1973 and the Food Regulations, administered by the Singapore Food Agency (SFA). Food products imported into Singapore can only be labeled in English. Key labeling information include product name, ingredient information, allergy information, volume, and manufacturer information. The label also indicates nutritional information, country of origin, expiration date, sweetener, low-calorie, and no sugar.

#### 3.2.5. Singapore Sanitary and Quarantine Standards

As Singapore imports approximately 90% of its food, securing safe food is important [62]. To this end, the SFA was established in 2019 to oversee food safety from farm to table The SFA integrated food-related functions previously performed by the Agri-Food and Veterinary AVS, National Environment Agency, and Health Sciences Authority. Food safety is an important global public health concern. Therefore, it is necessary to minimize the possibility of exposure to diseases through chemical or microbial contamination of food. In particular, heavy metals and antibiotics must be considered in the case of poultry.

Singapore’s meat product standards include microbial and chemical analyses (heavy metals, pesticide residues, and veterinary drug residues). The total plate count must meet the following criteria: N = 5, C = 3, m = 5.0 × 10^5^, M = 1.0 × 10^7^ CFU/g. Fecal *E. coli* must meet the following criteria: N = 5, C = 2, m = 1.0 × 10^2^, and M = 5.0 × 10^2^ CFU/g. Coagulase-positive *S. aureus* must meet the following criteria: N = 5, C = 2, m = 5.0 × 10^2^, M = 1.0 × 10^3^ CFU/g. *Salmonella* spp. met the following criteria: N = 5, C = 1, M = not detected in 25 g, and M = not detected in 25 g; *L. monocytogenes* met the following criteria: N = 5, C = 0, m = not detected in 25 g, and M = not detected in 25 g (Table 3). The limits of heavy metals in the chemical analyses must meet the following criteria: Mercury: 0.05 ppm; tin: 250 ppm, cadmium: 0.2 ppm; and antimony (1 ppm) (Table 4). The pesticide residues had a maximum of four contents, including carbaryl, coumaraphos, dichlorvos, pirimiphos-methyl 2, and veterinary drug residues have 55 contents (Table 7) [63].

#### 3.2.6. Organizations and Regulations of Halal Certification

1. Singapore Customs and Excise is a trade facilitation and revenue enforcement agency that builds trust and protects revenue in accordance with the relevant laws.
2. The SFA is a Singaporean agency that is responsible for food security. It plays a pivotal role in quarantining and inspecting imported food and enforcing a regulatory inspection system to ensure public health and food safety through laws such as the Sale of Food Act 1993 [64].
3. MUIS is the sole authority responsible for managing and regulating halal certification for food establishments in Singapore and overseeing halal certification in a systematic and comprehensive manner (Adaptive Strategy for Technology-Based Halal Tourism Development in Indonesia: Lessons from Singapore’s success/comparison of halal certification). The MUIS is also responsible for halal assurance, cleanliness, hygiene, and the standardization of materials in Singapore. It operates under a HalMQ, which encompasses the entire food supply chain, from sourcing and storage to production, logistics, sales, and marketing. Halal certification is controlled by MUIS under the AMLA, as described in Sections 88(1) and (2). The first part of these standards, MUIS-HC-S001, provides general guidelines for the handling and processing of halal foods. The second part provides general guidelines for the development and implementation of a Halal Quality Management System [26,65].

### 3.3. Indonesia

#### 3.3.1. Halal Certification in Indonesia

Indonesia is a predominantly Muslim country (87%) and has the world’s largest Muslim population (250 million people) [66]. Halal and Islamic kosher standards are important for most of the Indonesian population. The Indonesian Halal Certification Organization is BPJPH (BPJPH). The Indonesian Ministry of Religious Affairs established a new halal certification body called the Halal Product Certification Agency. Under the 2019 “Halal Product Assurance Law”, the LPPOM MUI transitioned from handling halal certifications to serving as a halal inspection body [67]. BPJPH oversees receiving and issuing certification applications. The LLPOM MUI conducts halal inspections, whereas the Indonesian Ulema Council (Majelis Ulama Indonesia, MUI), as the supreme Islamic clerical authority, retains the exclusive power to issue the final Fatwa (religious decree) that validates a product’s halal compliance, ensuring that all certified products strictly adhere to Islamic law through its final religious evaluation [68].

According to Law No. 33 of 2014, Indonesia requires imported meat products (excluding pork) to be accompanied by a halal certificate issued by an accredited halal certification body. This law requires halal certification of all products sold in Indonesia, including food, beverages, pharmaceuticals, and cosmetics. Only halal-certified products can be sold in the market. Indonesia has laws regulating agricultural products, slaughterhouses, and dairy products in accordance with halal regulations.

The halal certification procedure in Indonesia, coordinated by BPJPH, follows a structured, multi-stage process involving several entities [69]. It commences with the applicant submitting an online application through the official BPJPH portal. Upon receipt, BPJPH conducts an initial document evaluation to assess completeness. Once the application passes this stage, BPJPH notifies an accredited Halal Inspection Agency (Lembaga Pemeriksa Halal, LPH) and requests a cost estimate for the inspection, which is then relayed to the applicant. The applicant then proceeds to make the requisite fee payment to BPJPH. The assigned LPH subsequently performs a detailed technical review (LPH review), which typically includes an audit of facilities, processes, and ingredients, and submits its report. Based on the LPH’s findings, the MUI conducts a religious review and issues a Fatwa (religious decree) determining the product’s halal status. Following a positive Fatwa, BPJPH enacts a formal halal decree (Keputusan Halal). The process culminates with BPJPH issuing the official halal certificate to the applicant.

In response to an outbreak of high pathogenicity avian influenza (HPAI), Indonesia implemented strict conditions for poultry imports, including the mandatory requirement for halal certification, starting in October 2024 [70]. Countries that export poultry to Indonesia must not have experienced an HPAI outbreak in the 12 months prior to export [71]. The laws and regulations governing the slaughter and distribution of halal poultry in Indonesia, including chickens and ducks, are primarily based on the Halal Product Assurance Law (Law No. 33/2014) enacted in 2014 [72,73]. Recently, Government Regulation No. 42 of 2024 (CPP No. 42/2024) [74] adjusted the validity period of halal certificates to permanent if the ingredients or processes have remained unchanged. The deadline for mandatory certification is applied differently based on the business scale, with micro and small businesses granted a grace period until 2026. The Indonesian National Standard (SNI) is strengthening sanitary and quality regulations for animal-based foods such as poultry meat. The SNI establishes quality and safety standards and manages aspects such as the permissible levels of microbial contamination in poultry products. Therefore, compliance with national standards is essential for halal certification. The core elements of Indonesia’s halal poultry regulations include the Halal Product Assurance Law 2014 and its implementation, the roles of halal certification bodies (BPJPH and MUI), strict Islamic slaughter procedures, and quality control through national standards. Poultry products must comply with these regulations to obtain halal certification to be distributed in Indonesia.

In Indonesia, applications for BPJPH halal certification must be submitted online (https://ptsp.halal.go.id/starting accessed on 5 February 2026). This system allows companies to manage halal applications or certificates and submit related documents. The required documents include the Business Registration Certificate, Halal Certification Application Form in the BPJPH format, the Halal Assurance System Internal Manual (HAS 23000) [27,75], the Product Production Procedure Flowchart, documentation proving the absence of pork, a list of production facility addresses, additional certificates (HACCP, ISO, GHP, and GMP), and a factory location map. Foreign companies must also provide a power of attorney for Indonesian importers and an apostille-certified English halal certificate. The BPJPH must review and verify its application within one business day of the application date.

#### 3.3.2. The Process for Registering and Quarantine

Indonesia has unique customs clearance (CC) procedures. Import duties and taxes must be paid before submitting an import declaration. According to Indonesia’s regulation 9/2024 regarding Quarantine Documents and Seals, exporters of animals and animal products must submit a prior notice of exports to the Indonesia Quarantine Agency (IQA). Additional information about the portal used for the prior notice, including the user manual, can be found on the relevant website (https://notice.karantinaindonesia.go.id/ accessed on 5 February 2026). The Ministry of Agriculture is aware that the IQA implemented these requirements on 6 October 2024. The IQA advised exporters to submit a prior notice for consignment as soon as possible. The Department of Agriculture, Fisheries and Forestry understands that prior discoveries cannot be amended once they are submitted. Exporters are encouraged to submit additional prior notices, as needed, to ensure that their consignment details are complete and accurate before products arrive in Indonesia.

Indonesia’s quarantine system follows Law No. 21 of 2019 [76] regarding the quarantine requirements for animals, aquatic products, and plants. The quarantine procedure follows the sequence of Inspection—Quarantine—Observation—Treatment—Detention—Refusal of entry—Disposal—Clearance.

Documents required for quarantine can be submitted online. Essential documents include: Certificate of Origin, Animal Health Certificate, Certificate of Sale or Ownership, Export Permit, Quarantine Authority documents, Import Permit, Pre-shipment Inspection Report, Taxpayer Identification Number; and Customs Registration Number or NIB (Nomor Induk Berusaha, the Business Identification Number).

All products must also be registered with the Indonesian Food and Drug Authority before they can be distributed throughout Indonesia. The BPOM is Indonesia’s primary regulatory agency responsible for overseeing food, pharmaceutical, and public health. As of 17 October 2019, the BPOM officially enforced mandatory halal certification for all products (2014 No. 33 Halal Product Assurance (JPH), Article 67(1) of the Halal Product Assurance Law (JPH), Article 4 of JPH, and Articles 1(1) and 1(2) of Law No. 1) [77,78].

#### 3.3.3. Indonesia Import Procedure

Import procedures are based on quality and include halal standards, national standards, and Indonesian Food and Drug Authority certifications. All imported goods must obtain a Foreign Food Registration Number and BPOM certification to apply for import permits for shipment to Indonesia [79]. Indonesia enforces an SNI system that restricts customs clearance for products that have not previously obtained SNI certification. This is mandatory, and an SNI certificate must be attached during customs clearance.

Indonesia’s import clearance procedure generally follows the following sequence: preparation of essential import clearance documents, confirmation of tariff rates and preparation of the import declaration, payment of import duties, transmission of the import declaration and notification of acceptance results, and clearance after cargo inspection. For import clearance, particularly for poultry, which is subject to strict food hygiene and animal quarantine requirements, additional documents are necessary, such as the Indonesian Food and Drug Authority certification and quarantine certificates.

The required documents are as follows:

- Original bill of lading & delivery order;
- Invoice & packing list;
- Insurance policy;
- Form AK (when issued);
- Sales contract;
- Purchase order;
- Remittance slip (proof of payment for goods)/bank statement (if requested by customs);
- Catalog or specifications.

Furthermore, import certification numbers and licenses can be applied for and issued through the NIB. Customs classify importers into three tiers—Priority, Green, and Red—by applying differentiated clearance procedures. Poultry requires quarantine-related recommendations, international standard certifications (GMP, HACCP, and ISO 22000), and certificates of origin [80].

#### 3.3.4. Indonesia Food Labeling Format

In Indonesia, labeling regulations are governed by Decree No. 278 [81] of the Food and Drug Administration regarding plans to establish and amend related laws and regulations. Labels for processed foods distributed in Indonesia must include the following information: product name, ingredient list, net weight or volume, manufacturer/importer information, halal marking (if needed), production or date code, use-by date, BPOM registration number, and source of certain food items. Labels must be written in Bahasa Indonesia using Arabic numerals and Roman text. Foreign terms can be used if there are no Bahasa equivalents. Furthermore, background images, colors, or decorations that make the main label’s text difficult to read should be avoided. Nutritional labeling guidelines are based on BPOM Regulation No. 22/2019 [82]. All processed foods must display nutritional value information on their labels. Labeling requirements for processed foods with additives must provide key information, including the group name of the additive, specific name of the additive, and its registration number.

Imported product controls have been strengthened, requiring the attachment of overseas factory information and hygiene certificates. Therefore, imported food products must be accompanied by information and hygiene certificates from overseas manufacturing plants. Furthermore, meat products that are considered high-risk products may undergo on-site inspections and are required to submit a PMR.

#### 3.3.5. Indonesian Sanitary and Quarantine Standards

Poultry is a vital source of animal protein. Protein is essential for human growth and development. This has led to a high demand for poultry products worldwide. Indonesia places great importance on ensuring the safety of chicken meat at every stage, from farm to fork. Indonesia rigorously manages food safety by participating with the government, industry, industry, and consumers [83,84].

Meat product standards in Indonesia include microbial and chemical analyses (heavy metals and pesticide residues). Veterinary drugs are regulated by the Ministry of Agriculture, Forestry, and Fisheries Regulation No. 14/Permentan/PK.350/5/2017, which prohibits the use of antibiotics as a feed additive for animals, including poultry. This regulation to halt the use of antibiotics was officially announced in 2017; however, ensuring compliance with these regulations remains challenging. The standards for fresh and frozen meat products in Indonesia include microbial and chemical analyses (heavy metals, pesticides, and veterinary drug residues). For microbial analyses, the total count in meat products must be lower than 10^4^ CFU/g. The coliform count must be lower than 10^2^ CFU/g, the *Escherichia* count must be lower than 5 × 10^1^ CFU/g, the *Enterococcus* count must be lower than 10^2^ CFU/g, the *Staphylococcus aureus* count must be lower than 10^2^ CFU/g, and the *Salmonella* spp. count must be negative (Table 3).

In the chemical analyses, the limits for the heavy metals are mercury 1 ppm, lead 20 ppm (mg/kg), cadmium 5 ppm, and arsenic 5 ppm (Table 4). Pesticide residues have a maximum content of 122 including from 2,4, D to Trinexapac-ethyl. Veterinary drug residues have a maximum content of 15 (Table 8).

The Maximum Residue Limits (MRLs) of pesticides and other contaminants in food and fresh agricultural products are regulated by Law No. 18/2012 (“The Food Law”) in the chapter on food safety and quality [85].

#### 3.3.6. Organizations and Regulations of Halal Certification

1. Indonesian Quarantine Agency (IQA)

The IQA is the enforcement body for quarantine systems. The IQA implements integrated quarantine and monitoring in the fields of animal, plant, and aquatic products (fish). These measures ensure the safety of distributed goods, prevent the spread of pests and diseases, and enhance the efficiency of monitoring and quarantine implementation.

2. Organization of halal

Halal certification bodies include the MUI, Indonesia’s largest Muslim organization.

The MUI retains responsibility for determining whether a food product is halal, according to Sharia law. The MUI Fatwa oversees halal certification approvals through its Ethics Committee, and LPPOM-MUI conducts technical halal certification inspections. The BPJPH is the primary agency that oversees halal certification procedures in Indonesia. It is a department of the Indonesian Ministry of Religious Affairs and the only halal certification body in Indonesia with legal authority. For halal certification, the requirements and regulations are emphasized throughout the manufacturing process. HAS 23000:1 and HAS 23000:2 are the essential components of this system. These include the Requirements of Halal Certification (HAS 23000), Requirements of Halal Food Material (HAS 23201), Guidance on the Implementation of Halal Assurance System Criteria in the Processing Industry (HAS 23101), Halal Assurance System (HAS 23301), and Guidelines of Halal Assurance System Criteria on Slaughterhouses (HAS 23103) [86,87,88,89,90].

3. Ministry of Trade of the Republic of Indonesia (Ministry of Trade)

The Ministry of Trade enforces strict adherence to halal standards to secure public trust in food safety and quality and collaborates with the relevant agencies to ensure that the necessary halal certification requirements are met. Halal certification implies that compliance with halal regulations must be maintained throughout the value chain, including transportation and storage, as reflected in the export procedures managed by the Ministry of Trade.

Additionally, the agencies responsible for food imports include the Ministry of Industry, Ministry of Trade, and the Directorate General of Customs and Excise. Food control agencies comprise the Ministry of Food and Drug Safety, Ministry of Agriculture, and Ministry of Oceans and Fisheries.

### 3.4. Synthesis and Comparative Overview

A consolidated comparison of the key elements of the halal certification frameworks in Malaysia, Singapore, and Indonesia is presented in Table 9, which summarizes the certification logos, responsible organizations, core regulatory documents, and underlying standards.

## 4. Conclusions

This study underscores the increasing consumer demand for reliable halal products, necessitating robust halal guarantees to build trust in domestic and international markets. Employing an analytical method, we examined the legal frameworks governing halal systems in Indonesia, Malaysia, and Singapore—key players in the ASEAN halal sector. Our comparative analysis, as synthesized in the preceding section, revealed distinct national approaches: Indonesia’s mandatory certification, Malaysia’s predominantly voluntary system (with specific mandatory requirements for meat imports), and Singapore’s centralized state-managed framework. These differences are driven by unique regulatory bodies (e.g., BPJPH, JAKIM, MUIS) and reference standards (e.g., HAS 23000, MS 1500, MUIS-HC), significantly impacting the halal certification landscape and creating challenges for cross-border trade.

The observed variations in regulations, procedures, and auditing methodologies, while preserving local halal integrity, pose considerable barriers for businesses operating across these borders. Understanding these diverse national policies is thus paramount for market participants who wish to navigate the complexities of the global halal industry effectively. Such insight not only enhances confidence in halal food among all consumers but also promotes its wider inclusion in daily diets. This necessitates strategic adaptation by businesses and highlights the practical implications of our research for all stakeholders.

Based on our findings, we propose the following key strategies to foster a more integrated and efficient global halal market:

1. Mitigate Exporter Disruption: Address the most disruptive differences—divergent mandatory/voluntary statuses and specific technical requirements for raw material sourcing and auditing protocols—by developing mutual recognition agreements focused on core halal principles and common audit criteria. This will streamline cross-border processes and reduce compliance burdens, particularly for Indonesia’s broad mandatory scope.
2. Harmonize Standards for Efficiency: Achieve realistic minimal harmonization by standardizing the core definition of “halal” and common audit checklists for widely traded ingredients across MS 1500, MUIS-HC, and HAS 23000. Aligning on universally accepted critical control points can significantly reduce complexity and facilitate smoother trade.
3. Enhance Digital Transparency: Implement interoperable digital platforms for halal certification documentation across these nations. A common digital interface or data exchange protocol would enable streamlined application processes, improved traceability, and reduced administrative overhead by allowing single submissions and verification across authorities.
4. Invest in Capacity Building: Foster a more unified interpretation and consistent application of halal principles by investing in collaborative capacity-building programs for halal auditors and technical experts across ASEAN countries. This is especially crucial for complex products like pharmaceuticals and cosmetics.

Finally, we acknowledge the limitation of this study’s focus on three countries, which restricts the generalizability of its findings to the broader ASEAN region. Future research should expand the geographical scope of this work to include more ASEAN nations and employ robust methodologies such as systematic reviews of regulations and in-depth stakeholder interviews. This approach will provide a more comprehensive and practical understanding of halal standardization and regulatory harmonization, thereby facilitating integrated, policy-driven growth within the global ASEAN halal industry.

## Figures and Tables

**Table 1:** Core regulatory and standard documents analyzed in this review [25,26,27].

Country	Key Document/Standard	Issuing Authority	Key Version/Year
Malaysia	MS 1500:2019 Halal Food—Production, Preparation, Handling and Storage—General Guidelines	Department of Standards Malaysia (JSM)	2019
	Malaysian Halal Certification Procedure Manual	JAKIM	2020
	Food Act 1983 & Regulations 1985	Ministry of Health	1985 (Amended)
Singapore	MUIS Halal Standard: General Guidelines (MUIS-HC-S001)	Islamic Religious Council of Singapore (MUIS)	Current Version
	Sale of Food Act (Chapter 283) & Food Regulations	Singapore Food Agency (SFA)	Amended up to 2023
Indonesia	HAS 23000:1 Halal Certification Requirements—Halal Assurance System Criteria	LPPOM MUI/BPJPH	2012 (under revision)
	Law No. 33 of 2014 on Halal Product Assurance (JPH)	Government of Indonesia	2014
	Government Regulation No. 42 of 2024 on Implementation of Halal Product Assurance	Government of Indonesia	2024

**Table 2:** Documents required for Halal certification of slaughterhouses.

No.	Content
1	Company profile
2	Company registration certificate
3	Business/manufacturing license from local council
4	Business license
5	Name and product description/menu for verification
6	Type of packaging materials, Design and label of products
7	Contents of ingredients
8	Names and addresses of manufacturers/supplier of the ingredients
9	Halal status for the ingredients and the Halal certificate or the product specification for critical ingredients (if applicable)
10	Manufacturing process flow chart and production procedures
11	Other documents such as HACCP, ISO, GHP, GMP, TQM and so forth (if applicable)
12	Manufacturing license from Cosmetic and drug Control Authority (for health products and cosmetic)
13	Premise/factory location map
14	Layout plan
15	Copy of Import Permit Issued by the Dept of Veterinary Services Malaysia for meat/animal-based product
16	Copy of annual financial income statement
17	Copy of valid Halal certificate of ingredients or copy of product specification
18	Copies of identity cards and offer letter for two Muslims with Malaysian citizenship at the production area, or chef at the food outlet/premise or checker for the slaughter house
19	Slaughtering certificates for the slaughterer (for slaughter house only)
20	VHC from Veterinary Department for slaughter house (for slaughter house only)
21	Copy of expired Halal certification

**Table 3:** Regulation standard of microbiology.

	Malaysia	Singapore	Indonesia
Total count	10^6^ CFU/g	N = 5, C = 3, m = 5.0 × 10^5^, M = 1.0 × 10^7^ CFU/g	10^4^ CFU/g
Coliform	5 × 10^1^ CFU/g	-	1 × 10^2^ CFU/g
*Escherichia coli*		N = 5, C = 2, m = 1.0 × 10^2^, M = 5.0 × 10^2^ CFU/g	5 × 10^1^ CFU/g
*Salmonella* spp.		N = 5, C = 1, m = not detected in 25 g, and M = not detected in 25 g	Negative
*Staphylococcus aureus*		N = 5, C = 2, m = 5.0 × 10^2^, M = 1.0 × 10^3^ CFU/g	1 × 10^2^ CFU/g
*Listeria monocytogenes*		N = 5, C = 0, m = not detected in 25 g, and M = not detected in 25 g	
*Enterococcus* sp.			1 × 10^2^ CFU/g

N = number of sample units tested; C = maximum allowable number of defective sample units (with counts between m and M); m = threshold value for microbial count; M = maximum value for microbial count.

**Table 4:** Heavy metal regulation (mg/kg).

List	Malaysia mg/kg	Singapore	Indonesia
Lead	0.5		20
Cadmium	1	0.2	5
Arsenic	1		5
Tin	40	250	
Antimony	0.05	1	
Copper	1		
Mercury		0.05	1

**Table 5:** Veterinary drug residues in Malaysia (µg/kg).

No.	List	Muscle	Liver	Kidney	Fat	Tissue
1	Amprolium	500	1000	1000		
2	Avoparcin	100				
3	Colistin	150		200		
4	Danofloxacin	300	1200	1200	600	
5	Dihydrostreptomycin	500	500	1000	500	
6	Dimetridazole	5				
7	Doxycycline	100	300	600	300	
8	Enrofloxacin	30	30	30		
9	Erythromycin	300				
10	Ethopabate	500				
11	Flubendazole	200	500			
12	Flumequine	50	100	300		
13	Levamisole	10	100	10	10	
14	Maduramicin	240	720		480	
15	Neomycin	500	500	1000	500	
16	Nicarbazin	4000	4000	4000		
17	Nystatin					
18	Oxytetracycline	100	300	600	10	
19	Penicillin					
20	Robenidine hydrochlorine				200	100
21	Salinomucin	100				
22	Sarafloxacin		10		10	
23	Spectinomycin	300	5000	5000	2000	
24	Spiramycin	200	600	800	300	
25	Streptomycin	500	500	1000	500	
26	Sulfadimethoxine	100				
27	Sulfadimidine	100				
28	Sulfamethazine					100
29	Sulfaquinoxaline	100				
30	Tetracycline	100	300	600		
31	Tilmicosin	100			100	
32	Trimethoprim	50				
33	Tylosin	200	200	200	200	
34	Virginiamycin	100	300	500	200	

**Table 6:** Authentication scheme types in Singapore.

Operation	Content
Eating Establishment Scheme	This is issued to retail food establishments, such as restaurants, school cafeterias, and bakeries, as well as to temporary venues, such as markets, flea markets, and fairs.
Endorsement Scheme	Issued to imports, exports, or re-exports.
Food Preparation Area Scheme	Issued to food preparation and kitchen facilities.
Poultry Abattoir Scheme	This is issued to poultry slaughterhouses for fresh poultry.
Product Scheme	This is also issued for products manufactured or partially manufactured in Singapore.
Storage Facility Scheme	This is also issued to warehouses and mobile storage facilities.
Whole Plant Scheme	This is also issued to manufacturing facilities.

**Table 7:** Veterinary drug residues in Singapore (µg /kg).

No.	List	Kidney	Liver	Muscle	Fat/Skin
1	Ampicillin	50	50	50	
2	Avilamycin	200	300	200	200
3	Bacitracin	500	500	500	
4	Chlortetracycline and 4-Epichlortetracycline	1200	600	200	
5	Ciprofloxacin and Enrofloxacin	300			
6	Clopidol	20,000	20,000	5000	5000
7	Cloxacillin	300	300	300	
8	Colistin	200	150	150	150
9	Cyhalothrin	20	20	20	
10	Cypermethrin	50	50	50	
11	Danofloxacin	400	400	200	100
12	Deltamethrin	50	50	30	500
13	Diclazuril	2000	3000	500	1000
14	Difloxacin		1900		
15	Dihydrostreptomycin and Streptomycin	1000	600	600	600
16	Erythromycin-Chicken			100	
17	Erythromycin-Poultry	100	100		100
18	Florfenicol	750	2500	100	
19	Flubendazole	300	500	200	
20	Flumequine	3000		500	1000
21	Gentamicin	100	100	100	100
22	Halofuginone			10	20
23	Josamycin	40	40	40	
24	Kanamycin	2500		100	
25	Lasalocid	600	1200	400	600
26	Levamisole	10	100	10	10
27	Lincomycin	500		200	100
28	Maduramycin		720	240	
29	Monensin	10	10	10	100
30	Nafcillin	300	300	300	
31	Narasin	15	50	15	50
32	Neomycin	10,000	500	500	500
33	Nicarbazin	4000	4000	4000	4000
34	Norfloxacin-Chicken	20	20	20	
35	Norfloxacin-Poultry				20
36	Novobiocin			1000	
37	Oleandomycin			150	
38	Ormetoprim	100	100	100	100
39	Oxytetracycline & 4-EpiOxytetracycline	1200	600	200	
40	Penicillin G	50	50	50	
41	Robenidine	100	100	100	200
42	Salinomycin	500	500	100	
43	Sarafloxacin	80	80	10	20
44	Spectinomycin	5000	2000	500	2000
45	Spiramycin	800	600	200	300
46	Tetracycline & 4-EpiTetracycline	1200	600	200	
47	Tiamulin-Chicken	100	1000		
48	Tiamulin-Poultry			100	100
49	Tilmicosin				250
50	Trimethoprim	50	50	50	50
51	Tylosin	100	100		100
52	Tylvalosin		50		50
53	Virginiamycin-Chicken	200	200	50	
54	Virginiamycin-Poultry				200
55	Zeranol	2	2	2	2

**Table 8:** Veterinary drug residues in Indonesia (µg/kg).

No.	List	Muscle	Liver	Kidney	Fat
1	Alvendazole	100	5000	5000	100
2	Benzylpenicillin/Procainebenzyl-penicillin	50	50	50	
3	Chlortetracycline/Oxytetracycline/Tetracycline	200	600	1200	
4	Danofloxacin	200	400	400	100
5	Deltamethrin	30	50	50	500
6	Diclazuril	500	3000	2000	1000
7	Dihydrostreptomycin/Streptomycin	600	600	1000	600
8	Flubendazole	200	500		
9	Levamisole	10	100	10	10
10	Neomycin	500	500	10,000	500
11	Nicarbazin	200	200	200	200
12	Sarafloxacin	10	80	80	20
13	Spectinomycin	500	2000	5000	2000
14	Spiramycin	200	200	300	300
15	Sulfadimidine	100	100	100	100

**Table 9:** Comparative analysis of halal certification and regulations across countries.

	Malaysia	Singapore	Indonesia
Symbol	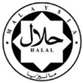	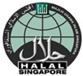	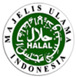
Site	www.islam.gov.my/halal (accessed on 5 February 2026)	https://www.muis.gov.sg/halal (accessed on 5 February 2026)	http://www.halalmui.org/ (accessed on 5 February 2026)
Organization	JAKIM (The Department of Islamic Development)	MUIS (Majlis Uama Islam Singapura)	MUI (Majelis Ulama Indonesia)
Regulation	MS 1500:2019: Halal Food: General guidelines for production, preparation, handling, storage [25] MS 1514:2009: Good Manufacturing Practice (GMP) for food [40] MS 1480:2007: Food Safety: Hazard Analysis and Critical Control Point (HACCP) system. Halal Supply Chain Standards [41] MS 2400-X:2019 (1,2,3): Halal Supply Chain Management System: General requirements for transport, warehousing, retail. Specific Product Standards [42] MS 2200-2:2013: Muslim-Friendly Consumer Products: General guidelines for the use of animal bones, hides, and hair [43] MS 2565:2014: Halal Packaging [44] MS 2627:2017: Detection of Porcine DNA: Test methods for food and food products [45] MS 2673:2021: Halal Assurance System: General requirements for establishing, maintaining, monitoring, and assuring compliance [46] Trade Descriptions Act 2011 & Related Regulations: Defines Halal criteria, designates JAKIM’s authority, mandates official Halal certification for products, regulates certification fees Food Act 1983 & Related Regulations: Food safety and hygiene control (including Food Regulations 1985) Animal Act 1953 & Related Legislation: Management of imported meat and slaughter (including Regulation of Animals 1962, Slaughterhouses Act 1993, Customs Act 1967)	MUIS-HC-S001General Guidelines for the Handling and Processing of Halal Food [26] MUIS-HC-S002: General Guidelines for the Development and Implementation of Halal Quality Management System [65] Administration of Muslim Law Act (AMLA) Section 88A [55] Singapore Muis Halal Quality Management System (HalMQ) [57]	HAS23000: Requirements of Halal Certification [27] HAS23201: Requirements of Halal Food Material HAS23301: Halal Assurance System [87] HAS23101: Guidance on the Implementation of Halal Assurance System Criteria in the Processing Industry [88] HAS23103: Guidelines of Halal Assurance System Criteria on Slaughterhouses [89] Government Regulation No. 42 of 2024 concerning the Implementation of Halal Product Assurance [72] Law No. 33 of 2014 concerning Halal Product Assurance (JPH) [77]

## Data Availability

No new data were created or analyzed in this study.
